# Automated Analysis of Cerebrospinal Fluid Cells Using Commercially Available Blood Cell Analysis Devices—A Critical Appraisal

**DOI:** 10.3390/cells10051232

**Published:** 2021-05-18

**Authors:** Manfred Wick, Catharina C. Gross, Hayrettin Tumani, Brigitte Wildemann, Martin Stangel, on behalf of the German Society of CSF Diagnostics and Clinical Neurochemistry, DGLN e.V.

**Affiliations:** 1 Institute for Laboratory Medicine, Ludwig-Maximilians-University Hospital, 81366 München, Germany; manfred.wick@googlemail.com; 2 Department of Neurology with Institute of Translational Neurology, University and University Hospital Münster, 48149 Münster, Germany; Catharina.Gross@ukmuenster.de; 3 CSF Laboratory, Department of Neurology, University of Ulm, 89081 Ulm, Germany; Hayrettin.tumani@rku.de; 4 Molecular Neuroimmunology Group, Department of Neurology, University Hospital Heidelberg, 69120 Heidelberg, Germany; Brigitte.Wildemann@med.uni-heidelberg.de; 5 Clinical Neuroimmunology and Neurochemistry, Department of Neurology, Hannover Medical School, 30559 Hannover, Germany

**Keywords:** cerebrospinal fluid, cell differentiation, automation, CSF diagnostics

## Abstract

The analysis of cells in the cerebrospinal fluid (CSF) is a routine procedure that is usually performed manually using the Fuchs–Rosenthal chamber and cell microscopy for cell counting and differentiation. In order to reduce the requirement for manual assessment, automated analyses by devices mainly used for blood cell analysis have been also used for CSF samples. Here, we summarize the current state of investigations using these automated devices and critically review their limitations. Despite technical improvements, the lower limit for reliable leukocyte counts in the CSF is still at approximately 20 cells/µL, to be validated depending on the device. Since the critical range for clinical decisions is in the range of 5–30 cells/µL this implies that cell numbers < 30/µL require a manual confirmation. Moreover, the lower limit of reliable erythrocyte detection by automated devices is at approximately 1000/µL. However, even low erythrocyte numbers may be of clinical importance. In contrast, heavily hemorrhagic samples from neurosurgery may be counted automatically at an acceptable precision more quickly. Finally, cell differentiation by automated devices provides only a rough orientation for lymphocytes, granulocytes and monocytes. Other diagnostically important cell types such as tumor cells, siderophages, blasts and others are not reliably detected. Thus, although the automation may give a gross estimate sufficient for the emergency room situation, each CSF requires a manual microscopy for cytological evaluation for the final report. In conclusion, although automated analysis of CSF cells may provide a first orientation of the cell profile in an individual sample, an additional manual cell count and a microscopic cytology are still required and represent the gold standard.

## 1. Introduction

The analysis of the cell number and the cell types in the cerebrospinal fluid (CSF) are important investigations in the routine workup when a neuroinflammatory disease is suspected clinically, as well as in subarachnoid hemorrhage and neoplastic meningitis. The analysis is also part of the diagnostic procedure in emergency medicine when a meningitis or encephalitis is suspected and thus, the technique has to be available 24/7. The gold standard has been the manual cell count, i.e., in a Fuchs–Rosenthal chamber. However, this requires the availability of specialized personnel. Thus, it has been attempted in the past to use commercially available blood cell analysis devices instead.

The analytical performance of these devices depends both on the analytical principles and the software used. Some manufacturers use for blood and body fluids such as CSF a combination of flow cytometry, mainly for nucleated cells, and impedance, for erythrocytes and platelets [1,2,3,4,5,6,7,8,9,10,11,12]. Other units are primarily designed for urine flow cytometry [6,10,13,14]. Flow cytometric measurements may be aided by fluorescent staining of DNA [1,2,3,4,5,6,7,8,9], peroxidase reactions by myeloperoxidase in myelomonocytic cells [15,16,17] or a more sophisticated multiangle polarized light scatter analysis [11], which enable at least a rough orientation of different nucleated cell types, especially leukocytes. Impedance (electrical resistance) measurements can only distinguish different corpuscular elements according to their size, as well as their swelling and shrinking properties with different reagents [7,18,19], with little leukocyte differentiation possible. Semiautomated image analysis, however, may provide a more differentiated analysis of cell composition [12].

Although these devices are available in many laboratories they are not optimized for the analysis of samples comprising low cell numbers, such as the CSF. Despite this caveat, their application for CSF samples has been investigated and these devices have been used in clinical care in order to optimize the availability of this investigation without the need for specialized personnel. Here, we review the evidence available for the use of automated CSF cell analysis and discuss the reproducibility of and pitfalls in routine application.

## 2. The Challenge

The challenge for automated CSF cell quantification is a reliable cut-off at 5 cells/µL, as this is the threshold value for a normal (<5 leukocytes/µL) and a pathological result. If such a differentiation cannot be achieved the practicability in daily clinical use is questionable, because clinicians rely on these values and treat patients accordingly.

Commercially available blood cell analyzers are generally not optimized for the investigation of samples with low cell numbers or CSF with abnormal cells. Nevertheless, their use in routine clinical application has been investigated for more than a decade [20,21,22,23] and some laboratories have used these systems even in routine clinical care. When applied in clinical practice, two different aspects of the analysis must be considered: (i) cell and erythrocyte count on the one hand and (ii) cell differentiation on the other. Furthermore, the applied technique also influences the analytical performance: optical or flow cytometric measuring systems offer more options for the analysis of nucleated cells with the possibility of fluorescent dyes or enzymatic staining, as compared to impedance measurement alone [24]. Both legal requirements for medical devices and local regulations such as the German Rili-BÄK (Richtlinie der Bundesärztekammer zur Qualitätssicherung laboratoriumsmedizinischer Untersuchungen—Guideline of the German Medical Association for quality assurance of medical laboratory examinations) must also be respected. While individual optical systems have US FDA approval for CSF at least above the respective quantification limit (e.g., >50 cells/µL, >10,000 erythrocytes/µL) [22,24], a CE certification is sufficient in the European Union. In case such certifications are not available for all measuring ranges, the operator is obliged to establish and validate internal specifications (“in-house method”). For cell counts, a distinction must be made between the absolute detection limit (limit of detection) and the functional detection limit or the limit of quantification as compared to the reference method chamber counting [25]. International requirements for functional sensitivity (variability coefficient, CV < 20% without taking bias into account) or quantification limit (CV < 20% with limited bias) are less strict than the common German definition of a quantitative method (CV < 15%). The quantification limits for automated systems are usually even higher than the upper limit of the reference range of <5/µL.

## 3. CSF White and Red Blood Cell Count

Sufficient sensitivity, if at all, can only be achieved by counting larger CSF volumes and thus absolute cell numbers in the modified so-called body fluid mode or CSF program [22,23]. Not only linearity is required for quantification, but also sufficient precision (VC < 15%), in particular in the lower measuring range. This also applies for the Fuchs–Rosenthal chamber, in which in low-cell samples, <20 cells/µL, the precision can only be achieved when the entire chamber is counted. Due to a lack of precision and linearity in the low cell count range, automated counting devices generally have a higher limit of quantification that still is, despite improvements, currently at approx. 20 cells/µL [24,26,27,28,29]. Older devices have sometimes an even higher limit of 50 cells/µL [11,19,21,22]. There is also a systematic upward deviation with false pathological assessments that would be particularly noticeable in the measurement range < 20 cells/µL if the reference range of < 5 cells/µL is not corrected upwards depending on the device [1,24,26,29,30]. In a comparative investigation of stabilized control samples in 10 round robin tests with numerous analysis systems that were commercially available at the time, it was found that the performance of devices with different measurement principles was generally in need of improvement [20]. The errors tend to increase with moderate blood contamination of the CSF [21,31,32], although the analysis of heavily bloody samples (>10,000 erythrocytes/µL) from neurosurgical samples with a simultaneously increased cell count (>20 leukocytes/µL) offers considerable practical advantages, with acceptable precision by means of some devices.

Even though technical and software improvements led to lower detection and quantification limits, including “research modes” [33,34,35], it was only possible to reduce the reliable quantification limit from 50 to 20 cells/µL. This is consistent with our own validations in the period 2005–2018 with a required CV of < 15% and limited bias. Below 20 cells/µL it was necessary to verify the result manually with a counting chamber (i.e., Fuchs–Rosenthal). This was particularly important for the management of patients in the emergency room who presented with the suspected diagnosis of a chronic inflammatory disorder. Once the cell number was within the reference range counted by an automated device (usually < 5 cells/µL) the results were more reliable, since there were hardly any false low values in this range [11,30] and the exact cell number is of less clinical importance when the cell count is normal. In case of doubt, however, every user must have validated this for his device himself. With higher CSF cell numbers (>50 cells/µL), on the other hand, the automatic cell counting can even be more precise and faster than the manual count due to the larger sample counted [29].

Regardless of the measuring range, particles other than leukocytes and erythrocytes can disturb the cell count. This can be recognized by interference in the scatter diagram [35,36], from pathogens such as bacteria (“ghost area”) and fungi, sometimes pollen, liposomes (“banana shape”), cell debris and cellular aggregates (uncontrolled scattering in the range of normal leukocytes or also in the high fluorescence range). In this case, grossly incorrect counting results are possible, which must be cross-checked in the manual counting chamber and by microscopic cell differentiation.

Even though the requirements for precision and sensitivity of the erythrocyte count are not as strict as for the CSF leukocyte count, small, invisible blood contaminations (<1000 erythrocytes/µL) should at least be semi-quantitatively detected. This is of great relevance for small or older subarachnoid hemorrhages. Furthermore, the erythrocyte number is required for the correction of artificially blood-contaminated CSF samples. While the quantification limit for older devices was 3000–10,000 erythrocytes/µL [11,22], it is now in the order of 1000/µL [26,30]. Lower values are, however, blocked by the manufacturer software, presumably due to poor precision. A manual chamber recount may also be required if the test strip screening is positive.

## 4. Cell Differentiation

**Figure 1:**
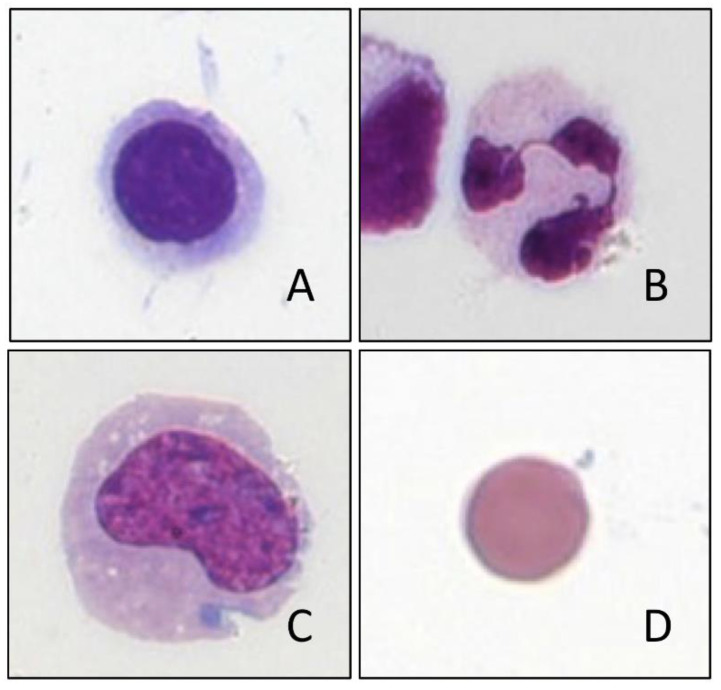
CSF cells correctly recognized by automated cell analysis: (**A**) lymphocyte, (**B**) granulocyte, (**C**) monocyte, (**D**) erythrocyte.

**Figure 2:**
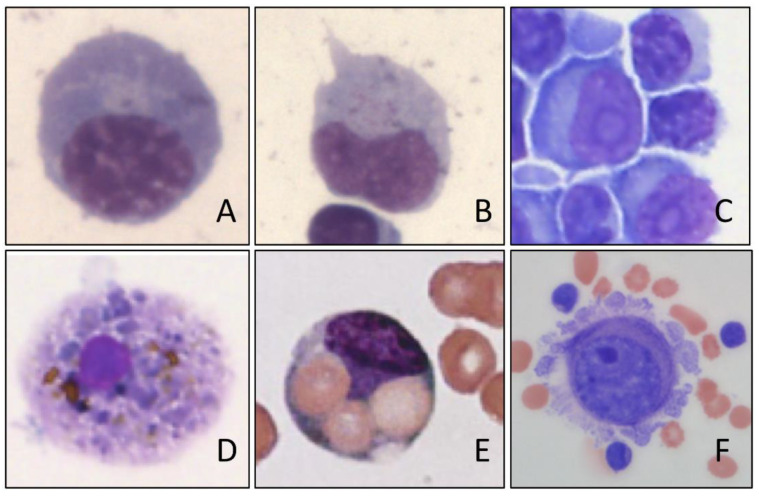
CSF cells not recognized by automated cell analysis: (**A**) plasma cell, (**B**) activated lymphocyte, (**C**) lymphoma cells, (**D**) siderophage, (**E**) erythrophage, (**F**) tumor cell.

While analysis systems that work exclusively with impedance measurements essentially assign the cells on the basis of size and swelling and shrinkage properties when various reagents are added, devices that measure optically or by flow cytometry allow potentially a more extensive cell differentiation based on the light scatter properties and fluorescent labeling (e.g., of the DNA or differential leukocyte lysis and cytochemical staining, e.g., based on myeloperoxidase) [24,37]. Thus, the standard evaluation allows at least a gross leukocyte differentiation in granulocytes and mononuclear cells in the CSF (Figure 1). In so-called research applications a more extensive differentiation may also be possible [33,38]. However, the distinction between granulocytes and mononuclear cells can already show a bias, depending on the number of cells [20,24,32]. The detection of highly fluorescent cells can indicate the presence of tumor cells, blasts, macrophages, siderophages, plasma cells, etc., but can also be disturbed by cell aggregates [35]. Due to this fact, tumor cells can neither be reliably detected nor excluded [3,38]. This is of special importance for CSF samples with unequivocal tumor cells despite a normal cell count [39]. Thus, such samples with the clinical suspicion of neoplastic meningitis and normal cell count are rather critical when analyzed by automated devices because both the cell number and the cell differentiation are at or below the absolute detection limit, even with modern analysis devices. False negative results would in these cases have fatal consequences for the patient. Apart from an initial orientation in acute inflammatory CNS diseases, automated CSF cell differentiation is therefore not recommended, because diagnostically relevant individual cells such as tumor cells, blasts, atypical lymphocytes, erythrocytes or siderophages, plasma cells, etc. (Figure 2), are neither sensitively nor reliably detected [3,21,36]. Thus, the gold standard for cell differentiation of CSF cells remains microscopy, possibly supplemented by immunophenotyping [40].

## 5. Conclusions

Despite technological improvements, the automated cell count and differentiation of CSF cells remains difficult. The detection limit of CSF cell counts depends on the device used and must be checked by the user on a case-by-case basis, in particular if the manufacturer does not provide sufficient validation data for the low cell count range. In the low pleocytosis range of 5–30 cells/µL, the inaccuracy of the automated cell count is generally the highest and requires a mandatory confirmation by a manual chamber count. This postulate is underpinned by the fact that this cell count range is the most critical for treatment and management decisions. On the other hand, heavily bloody samples with erythrocytes > 10,000/µL and nucleated cells > 20/µL may be counted at an acceptable precision more quickly, depending on the device. Gross counting errors may also occur due to particles other than cells or cell aggregates; however, this may be recognized by a disturbed scatter diagram.

The automated cytology and cell differentiation of CSF cells provides only a rough differentiation into granulocytes, lymphocytes and monocytes. Thus, it should only be used as a screening method for gross orientation. However, it requires further microscopy in order to detect cytopathological conditions including individual tumor cells, blasts, siderophages, plasma cells, activated lymphocytes and others (Figure 2).

In summary, automated analysis of CSF cells may give a first orientation, but has its limitations, in particular in the low cell count range and in the differentiation of CSF cells. Therefore, conventional cytology continues to be an integral part of CSF analysis and should be carried out regularly at each diagnostic lumbar puncture, irrespective of the total number of cells.

## Data Availability

Not applicable.
